# The Distribution of Phosphatidylinositol 4,5-Bisphosphate in Acinar Cells of Rat Pancreas Revealed with the Freeze-Fracture Replica Labeling Method

**DOI:** 10.1371/journal.pone.0023567

**Published:** 2011-08-15

**Authors:** Nami Ozato-Sakurai, Akikazu Fujita, Toyoshi Fujimoto

**Affiliations:** Department of Anatomy and Molecular Cell Biology, Nagoya University Graduate School of Medicine, Nagoya, Japan; University of Hong Kong, Hong Kong

## Abstract

Phosphatidylinositol 4,5-bisphosphate [PI(4,5)P_2_] is a phospholipid that has been implicated in multiple cellular activities. The distribution of PI(4,5)P_2_ has been analyzed extensively using live imaging of the GFP-coupled phospholipase C-δ1 pleckstrin homology domain in cultured cell lines. However, technical difficulties have prevented the study of PI(4,5)P_2_ in cells of *in vivo* tissues. We recently developed a method to analyze the nanoscale distribution of PI(4,5)P_2_ in cultured cells by using the quick-freezing and freeze-fracture replica labeling method. In principle, this method can be applied to any cell because it does not require the expression of artificial probes. In the present study, we modified the method to study cells of *in vivo* tissues and applied it to pancreatic exocrine acinar cells of the rat. We found that PI(4,5)P_2_ in the plasma membrane is distributed in an equivalent density in the apical and basolateral domains, but exists in a significantly higher concentration in the gap junction. The intracellular organelles did not show labeling for PI(4,5)P_2_. The results are novel or different from the reported distribution patterns in cell lines and highlight the importance of studying cells differentiated *in vivo*.

## Introduction

Phosphatidylinositol 4,5-bisphosphate [PI(4,5)P_2_] has been known to function in diverse phenomena, including membrane trafficking, cell polarization, ion channel regulation, and cytoskeletal assembly. PI(4,5)P_2_ is also the precursor of inositol-1,4,5-trisphosphate, phosphatidylinositol 3,4,5-trisphosphate [PI(3,4,5)P_3_], and diacylglycerol, which all play critical roles in signal transduction [1]. To understand how and where PI(4,5)P_2_ exerts its multiple functions, we must define the distribution of PI(4,5)P_2_ on the smallest possible scale using quantitative methods.

Live imaging using the GFP-tagged pleckstrin homology (PH) domain of phospholipase C (PLC)-δ1 as a probe has greatly advanced our understanding of the functionality of PI(4,5)P_2_ [2]. However, several potential problems are associated with the GFP-PH method [3], [4], including the perturbation of signaling by the expression of GFP-PH per se, the potential difficulty of GFP-PH to detect PI(4,5)P_2_ that is bound to effector proteins, and the influence of inositol-1,4,5-trisphosphate, which has a higher affinity for GFP-PH than PI(4,5)P_2_. Furthermore, the GFP-PH imaging method cannot be readily applied to cells *in vivo* because efficient transfection of GFP-PH cDNA is not easy.

Alternatively, the distribution of PI(4,5)P_2_ may be examined using conventional immunolabeling techniques [5], [6]. Although immunolabeling is a powerful method, the artificial redistribution of PI(4,5)P_2_ that may occur during the labeling procedure is a potential problem, especially for accurately examining PI(4,5)P_2_ localization at the small scale. This is mainly because aldehyde fixatives that are used for sample preparation do not react with most membrane lipids, and therefore, membrane fluidity persists even after fixation [7], [8].

To circumvent the fixation problem, we recently developed a physical method to fix membrane lipids using quick freezing and freeze-fracture replica formation [9]. In this method, lateral motion of membrane lipids is instantaneously stopped by freezing, and the molecules are then permanently immobilized by the evaporation of carbon and platinum. Membrane lipids trapped in the freeze-fracture replica can withstand harsh treatment with warmed SDS solution to remove extramembrane materials [10]. Thereafter the stabilized lipids are labeled for electron microscopy. The quick-freezing and freeze-replica labeling (QF-FRL) method can theoretically be used to examine the distribution of membrane lipids in any cell because it does not require the use of artificial probes or other types of cell manipulation.

By taking advantage of the versatility of the QF-FRL method, we examined the distribution of PI(4,5)P_2_ in rat pancreatic exocrine acinar cells in the current study. These cells form a simple epithelium with distinct apico-basolateral polarity and have well-developed intercellular junctions. With regards to the implication that PI(4,5)P_2_ is related to cell polarity formation [11], [12], we thought that the pancreatic acinar cell is an appropriate material to examine how PI(4,5)P_2_ distributes in the epithelium differentiated in a physiological environment. We modified the original QF-FRL method in several respects to analyze cells taken from *in vivo* tissues. By using the modified QF-FRL method, we examined the detailed PI(4,5)P_2_ distribution in the rat pancreatic acinar cell, and obtained several novel results as well as results different from those previously reported in cell lines in culture.

## Materials and Methods

### Ethics statement

All animal treatments in this study conformed to the Guidelines for Proper Conduct of Animal Experiments of Science Council of Japan and this study was approved by the Animal Experimentation Committee of Nagoya University Graduate School of Medicine (Approval ID: 23391).

### Probes

Mouse PLC-δ1 was used as a template to prepare recombinant GST-PH protein and its mutant version GST-PH(K30D,K32D), which does not bind to PI(4,5)P_2_ [13]. These probes were produced in *E. coli* and affinity-purified using a glutathione-agarose column as previously described [14]. The rabbit anti-GST antibody (Bethyl) and colloidal gold particles that were conjugated to protein A (The University Medical Center Utrecht) were purchased from their respective suppliers.

### Quick-freezing and freeze-fracture replica labeling (QF-FRL)

Pancreas was excised from adult male Wistar rats under anesthesia. The animals were fed ad libitum and thus the upper gastrointestinal tract contained digested food. Pancreas was minced with a razor blade to small pieces (∼1 mm^3^) in Tyrode's solution, placed on an aluminum disc (3 mm in diameter) with a shallow indentation (0.3 mm depth), and covered with a thin stainless steel foil (10 µm in thickness). The sample assembly was frozen using an HPM 010 high-pressure freezing machine (Leica) according to the manufacturer's instructions. The procedure from excision to freezing generally took at least several minutes. For freeze-fracture, the specimens were transferred to a cold stage of a Balzers BAF 400 apparatus and fractured at −130°C under a vacuum of ∼1×10^−6^ mbar. Replicas were made by electron-beam evaporation of carbon (C) (∼5 nm in thickness), followed by platinum (Pt) (2 nm) and then by C (20 nm) as described before [9].

Thawed replicas were treated with 2.5% SDS in 0.1 M Tris-HCl (pH 7.4) at 60–70°C for 30–60 min and digested with 0.25% trypsin and 0.5 mM EDTA in PBS for 1 h at 37°C. After washes with 1% Triton X-100 in PBS (PBST), the samples were treated with 100 µg/ml of DNaseI (Sigma) in a solution containing 50 mM Tris-HCl (pH 7.5), 1 mM MgCl_2_, and 0.01% BSA for 30 min at 37°C. After rinses, the replicas were treated again with 2.5% SDS in 0.1 M Tris-HCl (pH 7.4) at 60–70°C overnight. Following the treatment described above, replicas were stored in buffered 50% glycerol at −20°C until use.

### Replica Labeling

Freeze-fracture replicas were washed with PBST, blocked with a mixture of 3% BSA and 2% cold fish skin gelatin in PBS, and incubated with GST-PH (67 ng/ml) in 1% BSA and 1% cold fish skin gelatin in PBS at 4°C overnight. The samples were subsequently treated with rabbit anti-GST antibody (10 µg/ml) followed by colloidal gold (10 nm)-conjugated protein A (1∶60 dilution of the supplied solution), both for 30 min at 37°C in 1% BSA in PBS. After each incubation, replicas were washed extensively with 0.1% BSA in PBS. After brief rinses with distilled water, replicas were picked up on formvar-coated EM grids and observed with a JEM-1011 electron microscope operated at 100 kV. Digital images were captured by a CCD camera (Gatan Inc.) and subjected to further analysis. The binding specificity of the GST-PH probe in the labeling method was verified previously by using freeze-fracture replicas of liposomes containing different phosphoinositides [14].

### Statistical Analysis

Electron micrographs obtained from more than three independent experiments were used for the analysis. The areas of interest were measured using ImageJ (NIH), and the number of colloidal gold particles was counted manually. Statistical differences between samples were tested using the Student's *t*-test.

## Results

### Technical modifications to study cells of *in vivo* tissues

**Figure 1:**
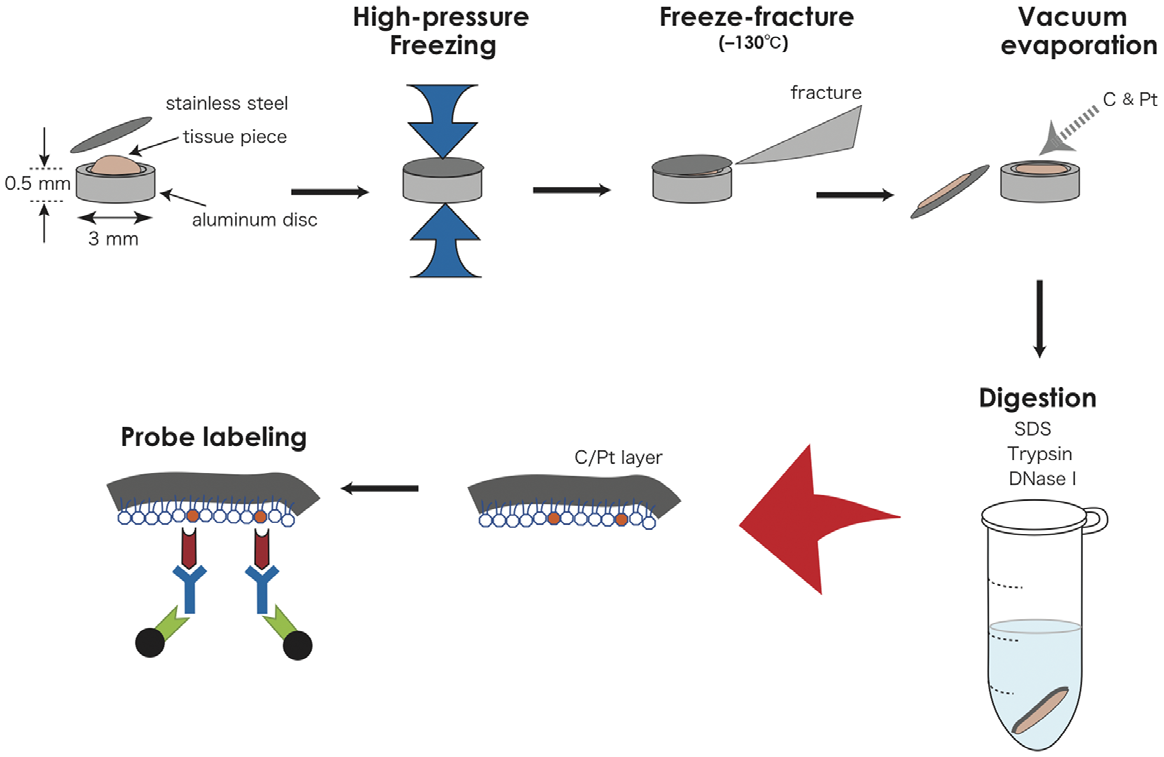
Outline of the modified QF-FRL method for the analysis of cells of *in vivo* tissues. A small piece of tissue was placed in an aluminum disc and covered with a thin stainless steel foil. The assembly was frozen using high-pressure freezing, and freeze-fracture replicas were prepared. The replicas were treated sequentially with trypsin, DNase I, and SDS and then labeled. PI(4,5)P_2_ was marked using colloidal gold particles and observed with electron microscopy.

The QF-FRL method has been successfully applied to cultured cells to observe distribution of PI(4,5)P_2_ and gangliosides GM1 and GM3 [14], [15], [16]. This method can be theoretically applied to any cell because it does not require any manipulation of the cell before freezing. But because tissue cells *in vivo* have several critical differences from cells in culture, we needed to modify the method in several respects (Fig. 1).

First, the cells cultured in a monolayer are usually less than 10–20 µm in vertical thickness, whereas tissue pieces are much thicker. Therefore, to freeze the samples without ice crystallization, we substituted the metal contact method for the high-pressure freezing method [17], [18], which can vitrify samples to a depth of 200 µm. The freezing rate is slower with the high-pressure freezing method (200°C/sec) than with the metal contact freezing method (∼10,000°C/sec) [19], but it should not be a problem unless analysis of molecular movements at the millisecond order is intended.

Second, the volume of cell and tissue fragments that adhere to the freeze-fracture replica is far greater in tissue pieces than that in cultured cells. The treatment in the SDS solution is not sufficient to remove all of the extramembrane materials from the replica of tissue specimens, which is problematic because the remnants tend to induce the artificial binding of probes. To efficiently remove extraneous materials, the replicas were cleaned using trypsin and DNase I before the SDS treatment. The trypsin and DNase I digestion did not affect the labeling of the membrane lipids, which was evaluated by comparing replicas of cultured cells with and without this treatment (data not shown).

Third, a mixture of cold fish gelatin and BSA rather than BSA alone was used to block the samples and dilute the GST-PH probe. The combination of the two proteins was effective in reducing non-specific labeling in replicas of *in vivo* tissue samples.

With these modifications, PI(4,5)P_2_ in the pancreatic acinar cell was labeled with sufficient specificity and intensity. The distribution density of colloidal gold particles that label PI(4,5)P_2_ in the basolateral plasma membrane of the epithelial cell was 303/µm^2^ when GST-PH was used at the concentration of 67 ng/ml, which was less than 422/µm^2^ in cultured human fibroblasts at the GST-PH concentration of 30 ng/ml [14]. The difference is likely to reflect the diversity in the actual PI(4,5)P_2_ concentration in the two cell types. We have observed that different types of cultured cells show highly variable labeling densities (A. Fujita and T. Fujimoto, unpublished observation).

At least several minutes were needed to dissect the pancreatic tissue from anesthetized rats and freeze it, but the following literature suggests that most *in vivo* properties are maintained: 1) Pancreatic acini obtained by dissection followed by extensive enzymatic treatments exhibits various physiological reactions [20]; 2) EM of isolated pancreatic acini [21] does not show notable differences from that of perfusion-fixed pancreas [22]; 3) With regards to PI(4,5)P_2_, a similar caveolar concentration was observed in smooth muscle dissected from mouse vas deference and in cultured human fibroblasts [14].

### PI(4,5)P_2_ does not show the apico-basolateral polarity but is concentrated in the gap junction

**Figure 2:**
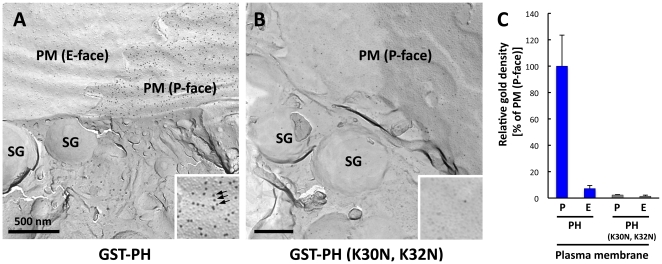
PI(4,5)P_2_ in rat pancreatic acinar cells labeled by the QF-FRL method. (A) PI(4,5)P_2_ was labeled positively only in the P face of the plasma membrane [PM (P-face)], whereas the E-face [PM (E-face)] was devoid of labeling. The P and E faces correspond to the inner and outer leaflet, respectively. Intracellular organelles, including secretory granules (SG), were not significantly labeled. The inset shows a small portion of the PM P face, and some gold particles are indicated with arrows. (B) A control experiment. GST-PH (K30N, K32N), a mutant that does not bind PI(4,5)P_2_, was used at the same concentration as GST-PH. GST-PH (K30N, K32N) produced little labeling. The inset is an enlargement of the PM P face to show the virtual absence of gold particles. (C) Quantification of the relative labeling density in the plasma membrane (average ± standard error). The density of colloidal gold particles is shown as the relative ratio to that of the P face labeled by GST-PH. The data were collected from three independent experiments, and areas greater than 7.5 µm^2^ were analyzed. The results show that only the P face is labeled specifically with GST-PH. The labeling density in the E face was not statistically different between samples treated with GST-PH and GST-PH (K30N, K32N).

In freeze-fracture electron microscopy, the inner (cytoplasmic) and outer (extracellular or luminal) leaflets of the membrane are represented as the P and E faces, respectively. The two faces are distinguished based on differences in the distribution density of intramembrane particles (i.e., it is higher in the P face than in the E face) and the ultrastructure of the surrounding areas (e.g., how many membrane leaflets exist between a leaflet to be identified and the extracellular space). In the pancreatic epithelium, the P face of the plasma membrane was densely labeled for PI(4,5)P_2_, whereas the E face was scarcely labeled (Fig. 2A). The labeling specificity was confirmed with a negative control experiment using a PH domain mutant. The mutant [GST-PH (K30N, K32N)] that contains 2 amino acid substitutions and lacks the binding capacity for PI(4,5)P_2_ [13] gave little labeling when used at the same concentration as the GST-PH (Figs. 2B, C).

**Figure 3:**
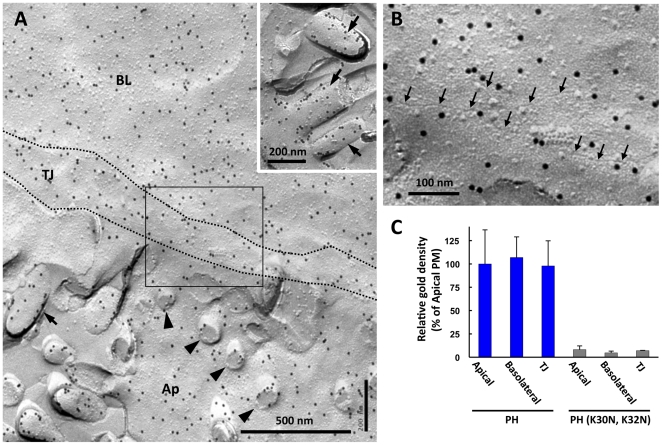
PI(4,5)P_2_ in the plasma membrane. (A) PI(4,5)P_2_ was labeled in an equivalent density in the P face of the apical domain (Ap), the basolateral domain (BL), and the tight junctional region (TJ) of the plasma membrane. The tight junctional region is demarcated by dotted lines. In the apical membrane, most microvilli were fractured at the basal portion and seen as stubs (arrowheads), but some were fractured longitudinally (arrows; arrows in the inset). The labeling density in the microvillar membrane was not significantly different from that in the flat portion of the apical membrane. (B) A high magnification image of the tight junctional region (the area in the rectangle of Fig. 3A). The tight junction was observed as shallow grooves in the P face (arrows). Some PI(4,5)P_2_ labeling was observed near the groove, but the total density in the junctional region was not different from that of the apical and basolateral membrane domains. (C) Quantification of the relative labeling density in the P face (average ± standard error). The density of colloidal gold particles is shown as the relative ratio to that of the apical domain. The data were collected from three independent experiments, and the total measured areas were 4.6 µm^2^ (apical), 8.6 µm^2^ (basolateral), and 7.7 µm^2^ (tight junction). The labeling density was equivalent in the three regions. GST-PH (K30N, K32N) produced little labeling in all three domains.

The pancreatic acinar cells comprise the highly polarized simple epithelium. The apical and basolateral domains of these cells are clearly demarcated by the well-developed tight junctions. The two membrane domains were distinguished unambiguously based on the tight junction and the apical microvillus in freeze-fracture electron microscopy. The tight junction in the quick-frozen specimens was observed as intermittent particles in the E face and as a shallow groove in the P face (Fig. 3, Fig. S1) [23].

The labeling density of PI(4,5)P_2_ in the P face did not show differences between the apical and the basolateral domains (Fig. 3). In the apical plasma membrane, most microvilli were observed as stubs that showed only the basal portion (arrowheads in Fig. 3A), but some microvilli were fractured longitudinally to show the distal portion (arrows in Fig. 3A and inset). In both instances, PI(4,5)P_2_ in the microvillar membrane was labeled at a similar density as that in the flat region of the apical membrane. The results indicate that the PI(4,5)P_2_ in the apical membrane distributed without distinct local concentration. The membrane in the tight junction area was also labeled in a similar density as the apical and basolateral membranes (Fig. 3B).

**Figure 4:**
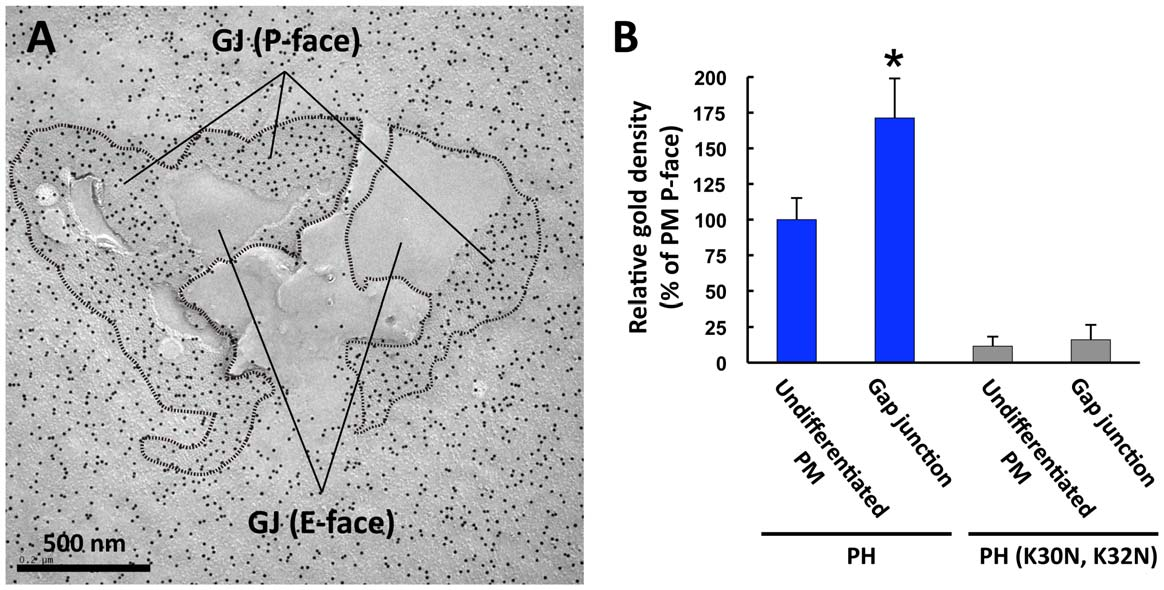
PI(4,5)P_2_ in the gap junction. (A) PI(4,5)P_2_ was labeled more intensely in the P face of the gap junction (GJ) than that of the surrounding undifferentiated basolateral membrane. The E face of the gap junction was not labeled. The gap junctional plaques are circumscribed by dotted lines. (B) Quantification of the relative labeling density in the P face (average ± standard error). The data were collected from pairs consisting of a gap junctional plaque and its surrounding membrane area, in four independent experiments. The labeling density in the gap junction is significantly higher than that of the undifferentiated membrane (Student's *t*-test, *p = 0.04). GST-PH (K30N, K32N) gave only negligible labeling both in the gap junction and in the surrounding membrane.

Gap junctions, which were identified unambiguously as plaques of densely-packed intramembrane particles in the P face, were labeled more densely than the surrounding basolateral membrane (Fig. 4, Fig. S2). The E face of the gap junction was devoid of labeling. Because the lipid-to-protein ratio in the gap junction is lower than that of the non-junctional membrane [24], the results suggest that the molar ratio of PI(4,5)P_2_ in the lipids of the inner leaflet is quite high in the gap junction.

### PI(4,5)P_2_ is not labeled in the intracellular organelles

The pancreatic acinar cells are also highly polarized in the disposition of intracellular organelles. Multiple layers of the rough endoplasmic reticulum (ER) and a cluster of large secretory granules are present in the basal and apical cytoplasm, respectively. This polarized disposition and characteristic morphology, which reflects the cellular exocrine activity, was used to identify the organelles in freeze-fracture replicas.

**Figure 5:**
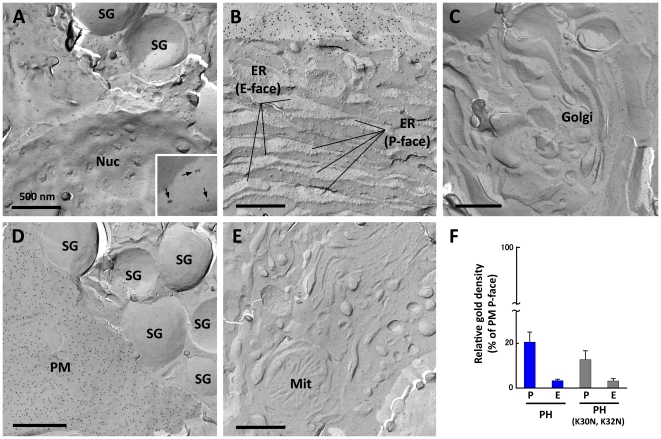
PI(4,5)P_2_ in intracellular organelles. (A-E) Organelles were identified by morphological criteria. The nuclear membrane (Nuc) (A), the ER (B), the Golgi apparatus (C), the secretory granule (SG) (A and D), and the mitochondrion (Mit) (E) were devoid of specific labeling for PI(4,5)P_2_. More colloidal gold particles were seen in the P face of the secretory granule than the other organelles, but they were observed in a similar density even when GST-PH was replaced with GST-PH (K30N, K32N) (Fig. 2B). The inset (A) is an enlargement of a small portion of the P face of the secretory granule membrane to show the presence of non-specific labeling (arrows). (F) Quantification of the relative labeling density (average ± standard error). The labeling density in the secretory granule is shown as the relative ratio to that of the plasma membrane. The data were collected from three independent experiments and areas more than 9.3 µm^2^ was measured. An equivalent number of colloidal gold particles were observed in the P face of the secretory granule when either GST-PH or GST-PH (K30N, K32N) was used. Therefore, we concluded that the labeling in the secretory granule was insignificant.

In more than 20 experiments we observed more than 300 cellular profiles that fractured across the cytoplasm and reveal various combinations of intracellular organelles; however, specific labeling for PI(4,5)P_2_ was not observed in any organelle in the P and E faces (Fig. 5). Organelles that were analyzed for PI(4,5)P_2_ labeling included the nuclear membrane (Fig. 5A), the ER (Fig. 5B), the Golgi apparatus (Fig. 5C), the secretory granule (Fig. 5D), and the mitochondrion (Fig. 5E). In the secretory granule membrane, gold particles were observed consistently in the P face; however, an equivalent density of gold particles was found even when GST-PH was replaced with GST-PH(K30N, K32N) (Fig. 5F). Therefore, we speculated that the observation in the secretory granule membrane was caused by nonspecific binding of a labeling probe(s) to an unknown component of the secretory granule membrane.

## Discussion

### Non-polarized distribution of PI(4,5)P_2_ in the plasma membrane

Intracellular distribution and dynamic movement of PI(4,5)P_2_ in cultured cells have been studied extensively using live imaging with PH domains that have been tagged with GFP [13], [25]. In Madin-Darby canine kidney (MDCK) cells forming cysts in three-dimensional culture, PI(4,5)P_2_ was observed to be enriched in the apical membrane [12], whereas PI(3,4,5)P_3_ was confined to the basolateral membrane [11]. Based on this observation along with other data, PI(4,5)P_2_ and PI(3,4,5)P_3_ were implicated as the apical and the basolateral membrane organizers, respectively [26].

In the present study, PI(4,5)P_2_ in the pancreatic acinar cells in the adult rat was detected in equivalent densities in the apical and basolateral membranes. This result may initially appear to contradict the findings in MDCK cells, but the difference may be explained in several ways. First, the MDCK model probably mimics a developing stage when the apical lumen is in the process of being formed. Therefore the patterns that are evident in the MDCK model may differ from those observed in the established epithelium in the adult rat tissue. Second, the pancreatic acinus and the MDCK cyst may use different mechanisms for apical lumen formation. Although the cord hollowing process in MDCK cells [27] also appears to be the predominant mechanism in the tubulogenesis of pancreas [28], the possibility remains that the terminal acinar portion may form by the process of budding from the polarized tubular epithelium [29]. Third, the GFP-PH signal observed in live imaging may primarily represent the PI(4,5)P_2_ population that is not bound to effector proteins, and may not reflect the density of total PI(4,5)P_2_. This is likely to happen because whether and to what extent GFP-PH replaces endogenous PI(4,5)P_2_-binding proteins has not been known. Therefore the PI(4,5)P_2_ density in the apical and basolateral membranes of MDCK cells may not be so divergent as suggested by live imaging.

All of the above possibilities are reasonable and are not mutually exclusive, but we think that the third possibility is the most plausible explanation of the results. Cdc42 is the master regulator of cell polarity [30], and the Cdc42-dependent recruitment of PTEN, which hydrolyzes PI(3,4,5)P_3_ to produce PI(4,5)P_2_, was previously identified as a critical event in the formation of the apical domain [12]. However, if we consider that the concentration of PI(4,5)P_2_ is as much as 1,000 times higher than that of PI(3,4,5)P_3_ [31], PI(4,5)P_2_ that is generated by PI(3,4,5)P_3_ hydrolysis constitutes only a very minor proportion of the total PI(4,5)P_2_ and should not have much significance [32]. The result indicates that unoccupied PI(4,5)P_2_ that is generated at the right location at the right time is more important in the cell polarization process than the total amount of PI(4,5)P_2_. To test this conjecture and elucidate the true PI(4,5)P_2_ distribution, it would be helpful to study the same cell system using both GFP-PH imaging and QF-FRL.

### Enrichment of PI(4,5)P_2_ in the gap junction membrane

The labeling for PI(4,5)P_2_ was more intense in the gap junction plaque than in the surrounding membrane. Because the lipid-protein ratio is lower in the gap junctional membrane than in the general membrane [24], the actual proportion of PI(4,5)P_2_ in phospholipids of the gap junction must be even higher than that indicated by the labeling density. This concentration of PI(4,5)P_2_ in the gap junction is intriguing, because cell-cell communication through the connexin 43 (Cx43)-based gap junction channel is regulated by the local concentration of PI(4,5)P_2_ [33], [34]. In Rat-1 fibroblasts, PLCβ3 shuts down the channel by hydrolyzing PI(4,5)P_2_ upon receptor activation [33].

The mechanism used by PI(4,5)P_2_ to regulate the gap junction is not known, but its direct binding to connexin molecules is a possible mechanism. Cx43 may bind to PI(4,5)P_2_ through a putative PI(4,5)P_2_-binding domain in the C-terminal domain, which contains positive charges that are interspersed with hydrophobic residues [33]. The gap junctions in the pancreatic acinar cells are composed of Cx32 and Cx26 and do not contain Cx43 [35]. However, Cx32 and Cx26 also contain a segment that is similar to the putative PI(4,5)P_2_-binding domain and may bind PI(4,5)P_2_ directly.

The abundance of PI(4,5)P_2_ in the gap junction suggests that the channel is in the open state in the pancreatic acinar cell observed in the present study. It would be interesting to study whether the density of PI(4,5)P_2_ changes when challenged with various reagents. The present method would be a useful tool for further analyses.

### PI(4,5)P_2_ in intracellular organelles

We observed more than 300 cross-fractured cell profiles, but no positive labeling was detected in any organelle. The absence of PI(4,5)P_2_ in the nuclear membrane, the ER membrane, and the mitochondrion was expected. However, the absence of PI(4,5)P_2_ labeling in the Golgi membrane was puzzling because the function of PI(4,5)P_2_ in membrane trafficking around the Golgi has been previously demonstrated [36], [37]. The phosphatidylinositol 4-phosphate 5-kinase activity was found in isolated Golgi [38], [39], and labeling for PI(4,5)P_2_ in the Golgi was observed in ultrathin cryosections [40]. On the other hand, however, a fluorescence microscopic study that demonstrated the presence of phosphatidylinositol 4-phosphate in the Golgi failed to detect PI(4,5)P_2_ [41].

The sensitivity of the current method should not be the cause of the negative results because as low as 0.5 mol% of PI(4,5)P_2_ in the liposomal membrane can be detected [14]. An obvious possibility is the variation between different cell types. Alternatively, the location of PI(4,5)P_2_ may have hampered its detection with the QF-FRL method. Because the plane of freeze-fracture seldom runs along membranes that are bent acutely, indentations or protuberances are not well represented in many samples. We speculate that if PI(4,5)P_2_ exists in the Golgi, it does not distribute evenly but is concentrated in a specialized compartment.

It is unclear why non-specific binding of GST-PH occurred in the secretory granule membrane. The PH domain of PLCδ1 has been used in many experiments, but non-specific binding has not been reported, as far as we know. However, most experiments have been done using isolated lipids and the possibility that the PH domain binds to certain non-lipid molecules is difficult to refute. This reinforces the importance of always running a control experiment using the GST-PH mutant.

### Concluding remarks

The present study demonstrated that the modified QF-FRL method can be successfully applied to cells taken from *in vivo* tissues to observe the distribution of PI(4,5)P_2_. The results showed that PI(4,5)P_2_ in the pancreatic acinar cells was evenly distributed in the apical and the basolateral membranes and that PI(4,5)P_2_ was locally concentrated in the gap junction. These results may be difficult to obtain using other methods available at present. It is always important to study cells that differentiate in a physiological environment. By expanding the applicability to other membrane lipids, the QF-FRL method may become an even more useful tool for such analyses.

## Supporting Information

Figure S1 **The tight junction.** The tight junction was observed as strands in the E face that separate the apical (Ap) and the basolateral (BL) membrane domains. The labeling for PI(4,5)P_2_ was observed in the P face of the plasma membrane. The apical domain is only observed in the E face.
(TIF)

Figure S2 **The gap junction.** The same micrograph as Fig. 4. The area in the rectangle of the left figure is enlarged to show the crystalline arrangement of intramembrane particles in the P face of the gap junction (GJ). The E face of the gap junction appears crystalline, but the dimples are not apparent in this micrograph.
(TIF)
