# Supplementary figures and images for: The Distribution of Phosphatidylinositol 4,5-Bisphosphate in Acinar Cells of Rat Pancreas Revealed with the Freeze-Fracture Replica Labeling Method
Source: PLoS One. 2011 Aug 15;6(8):e23567. doi: 10.1371/journal.pone.0023567 (PMC3156236; doi:10.1371/journal.pone.0023567)

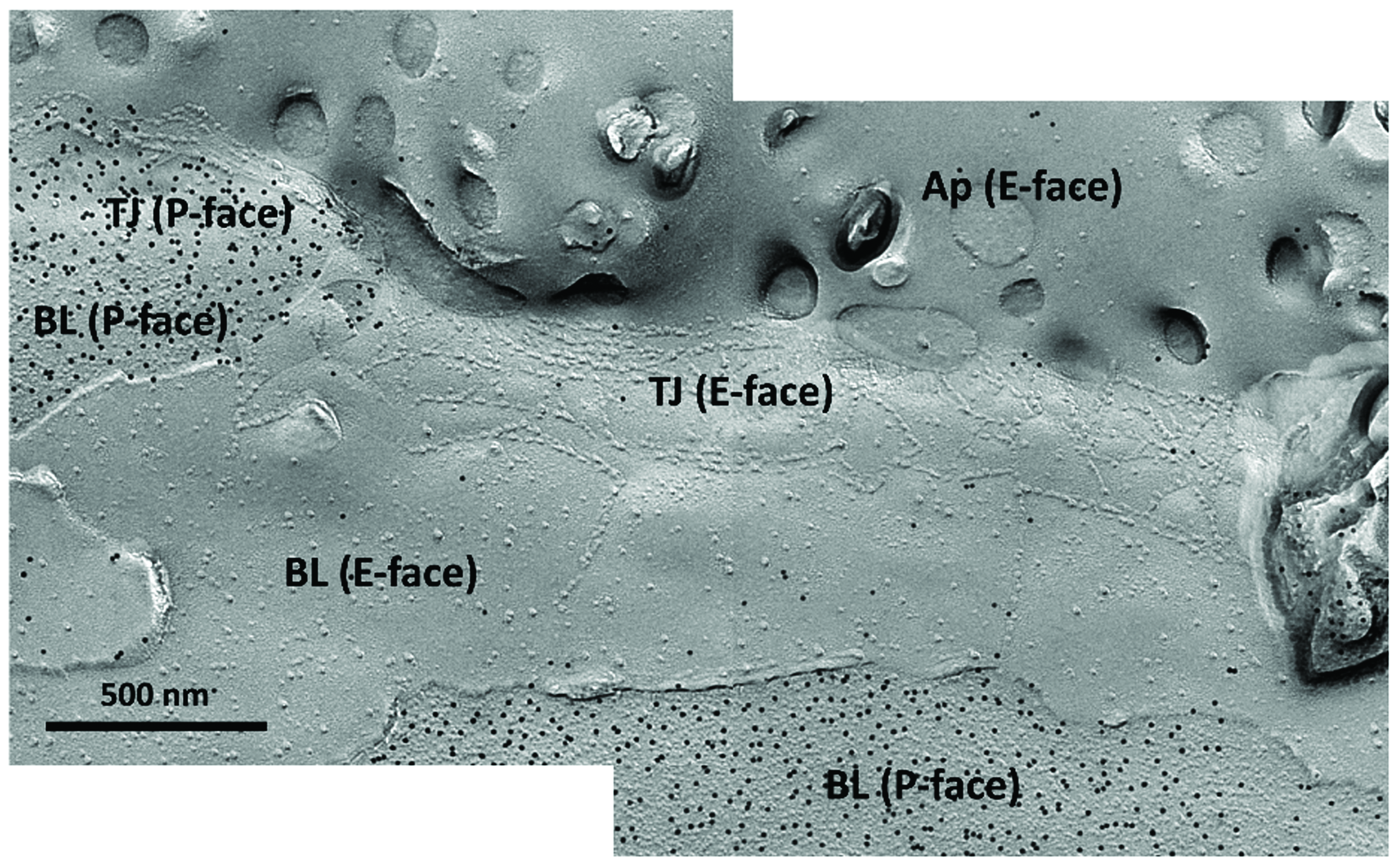

Supplement: Figure S1 — The tight junction. The tight junction was observed as strands in the E face that separate the apical (Ap) and the basolateral (BL) membrane domains. The labeling for PI(4,5)P2 was observed in the P face of the plasma membrane. The apical domain is only observed in the E face. (TIF) [file pone.0023567.s001.tif]

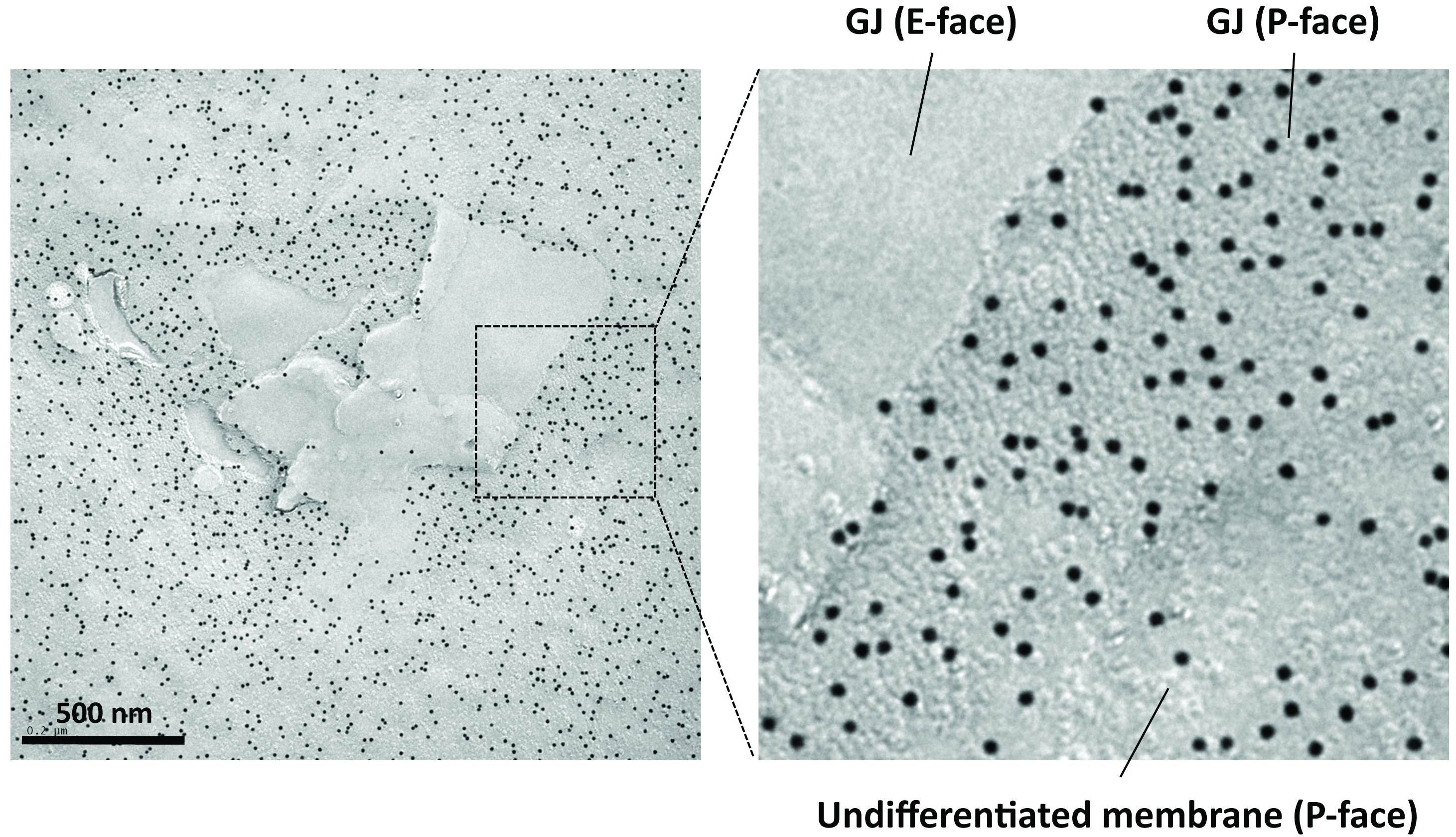

Supplement: Figure S2 — The gap junction. The same micrograph as Fig. 4. The area in the rectangle of the left figure is enlarged to show the crystalline arrangement of intramembrane particles in the P face of the gap junction (GJ). The E face of the gap junction appears crystalline, but the dimples are not apparent in this micrograph. (TIF) [file pone.0023567.s002.tif]
